# Habitat Selection of Wintering Birds in Farm Ponds in Taoyuan, Taiwan

**DOI:** 10.3390/ani9030113

**Published:** 2019-03-23

**Authors:** Chia-Hsuan Hsu, Jui-Yu Chou, Wei-Ta Fang

**Affiliations:** 1 School of Forestry and Resource Conservation, National Taiwan University, Taipei City 10617, Taiwan; d05625002@ntu.edu.tw; 2 Department of Biology, National Changhua University of Education, Changhua City 50007, Taiwan; jackyjau@cc.ncue.edu.tw; 3 Graduate Institute of Environmental Education, National Taiwan Normal University, Taipei City 11606, Taiwan

**Keywords:** habitat difference, irrigation ponds, landscape ecology, wintering birds

## Abstract

**Simple Summary:**

Identification of existing and potential irrigation ponds is essential for creating waterbird refuges to secure habitats for wintering waterbirds in anthropogenically influenced areas. In total, 45 ponds were surveyed in the Taoyuan Tableland in northwestern Taiwan. The association between pond dimensions and bird-species richness and community composition was determined by comparing the responses of functional groups to pond configurations. The results demonstrated that waterbirds, compared with landbirds, have a stronger correlation with pond variables. Our study provided substantial evidence that these artificial ponds had also influenced the distribution of wintering waterbirds.

**Abstract:**

Farm ponds or irrigation ponds, providing a vital habitat for diverse bird communities, are an environmental feature with characteristics that cross over typical urban and natural conditions. In this study, the species richness and community structure of irrigation ponds were characterized on the local and landscape scales. Within a landscape complex in the Taoyuan Tableland of Taiwan, 45 ponds were surveyed, ranging in areas from 0.2 to 20.47 ha. In total, 94 species and 15,053 individual birds were identified after surveying four times. The association between ponds and birds was determined to establish the effect of pond dimensions on species richness and community composition in the complex by comparing the responses of functional groups to pond configurations. Seven avian functional groups were identified. Compared with landbirds (i.e., families Alcedinidae, Apodidae, Icteridae, and Sturnidae), waterbirds (i.e., families Anatidae, Ardeidae, Charadriidae, Podicipedidae, and Scolopacidae) exhibited a stronger correlation with pond variables. Our study provides substantial evidence that these artificial ponds have influenced wintering waterbirds. The final results of this study may help stakeholders and land managers identify areas not to establish large-scale solar facilities considering waterbird habitats in pond areas.

## 1. Introduction

Over the years, human activities have severely affected wildlife and its habitat; therefore, it is crucial to balance the needs of people and wildlife. In particular, the habitats of birds are extensively exploited by humans through the use of land and water for agriculture, associated construction, and other types of development activities [1,2]. In Taoyuan Tableland, Taiwan, thousands of farm ponds have been constructed for irrigation. This area had more than 3290 ponds in the 1970s, but fewer than 1800 currently exist. Farm pond configurations and the complexity of their compositions exhibit marked diversity [3]. Pondscape was defined as “a series of water surfaces of ponds in association with various surrounding landforms, including farms, creeks, canals, roads, houses, woodlands, and other open spaces” [3,4,5]. Several migratory birds stop over at pondscapes. Because of their specific habitat requirements for stopover during migration, birds provide indicators regarding habitat conditions [6,7]. All avian species select a suitable habitat to ensure availability of food, water, shelter from the weather and predators and feasible nesting sites to reproduce. All species in a guild display similar characteristics. However, Duelli and Obrist suggested that generalist species might not be appropriate biodiversity indicators [8].

Anthropogenic influences (i.e., pollution, destruction, degradation, and other stress) can be monitored using bioindicators [9,10,11]. In this case, generalists are the ones that could benefit from a higher abundance of habitats that are spatially heterogeneous. However, specialists are the ones that thrive in a nearly homogenous habitat, with a high occurrence rate in their own specific habitat [12]. Thus, compared with generalists, specialists are less dependent on habitat scale and can only exist within a specific type of habitat. These species include waterfowl (Anatidae and Podicipedidae), shorebirds (Charadriidae and Scolopacidae), and wading birds (Ardeidae). They have specific habitat requirements and are generally unable to adapt to new diets or environmental conditions [13]. Therefore, specialists are more vulnerable than generalists to anthropogenic disturbance [14,15].

The guild concept involves the division of birds according to their habitats and further categorization according to landscape configurations. Root, the first avian scientist to propose the guild concept, defined a guild as “a group of species that exploit the same class of environmental resources in a similar way” [16]. He realized that the traditional taxonomic approach failed to categorize avian communities appropriately. For example, he described the “foliage-gleaning guild” as birds that obtain their food from foliage and occasionally from branches [16]. Thus, Root grouped five species with similar diets, foraging locations, and feeding behaviors into one guild [16].

After Root defined functional groups based on traditional guilds (considering diets and foraging strategies), other authors have followed his approach for investigating avian behavior and foraging strategies [17,18,19,20]. Studies have evaluated nesting, resting, singing, residential locations [3,21,22,23], foraging strategies, and singing locations [24]. However, most studies using functional groups have grouped species according to subjective criteria or a single behavior, focused on a single group or selected groups, at a single location or on small spatial scales [25,26].

Previous studies have also evaluated environmental conditions through examining guilds within heterogeneous landscapes [12,27]. We selected the definition of a guild that uses habitat preference to define functional groups [4]. Through categorization of birds as generalists or specialists, French and Picozzi [4] demonstrated that wintering birds were influenced by land use. Avian grouping aids in identifying avian diversity according to habitat, while tackling landscape complexity [28]. Because information is limited regarding environmental factors that affect avian guilds, previous avian studies have applied cluster analyses for the grouping of similar components of the avian community into respective functional groups [29,30,31]. The previous study aimed to identify groups of birds with certain habitat preferences by constructing groups (clusters) using multivariate data [30]. Both habitat- and landscape-scale avian community studies are required to understand habitat selection [32,33].

On a larger scale, landscape configurations account for variations in the richness and diversity of wintering bird species. An irrigation birds’ habitat can be evaluated according to the number of avian species it contains. Therefore, birds become a bio-indicator for different types of habitat [34,35]. Differences in edge disturbance affect birds differently on avian communities. To preserve biodiversity on different habitats and landscape scales, it is essential to understand the effects of different management strategies on diversity.

Avian ecologists have used guilds to avoid classified errors that can occur when considering a large number of species [36]. However, the taxonomic diversity of entire groups and specific guilds is well debated [37]. One drawback of using guilds is that the taxonomic approach to avian studies is not commensurate with landscape scales [37]. Furthermore, studies using aggregate species richness or diversity indices have often been oversimplified [38,39].

Although most of the farm ponds in Taoyuan are artificial, they provide food, refuge, and nesting sites for breeding birds. Thus, farm pond ecosystems in Taoyuan are crucial for these birds. Furthermore, farm ponds are wetlands for flood detention and water purification [40,41]. Farm ponds are vital for humans as well. However, current developments, such as large-scale solar facilities, in ponds tend to destroy pondscapes; therefore, we, ecologists and bird lovers, are actively trying to protect these ponds. This study includes data from 2003 to 2004; our goal was to present bird conditions before solar facility construction to inform decision-makers of the importance of ponds to wildlife. In this study, we compared approaches for calculating species diversity in specific functional groups, which helped select an approach for fitting avian communities in irrigation ponds. The purpose of this study was to identify bird guilds through cluster analysis, simply list the birds recorded, organize them into groups, and indicate those understood to be “generalists” or “specialists”. Therefore, this study aimed to (1) characterize and analyze the waterbird species around the irrigation ponds of Taoyuan and, (2) categorize their functional groups using cluster analysis, thereby grouping birds according to different habitats. We did not study avian feeding habits (i.e., insect feeding, seed feeding, algae feeding, fish or crustacean feeding) and their food; we instead focused only on the correlation between guild (i.e., species richness and individuals) and pondscape variables.

## 2. Materials and Methods

### 2.1. Study Site

**Figure 1:**
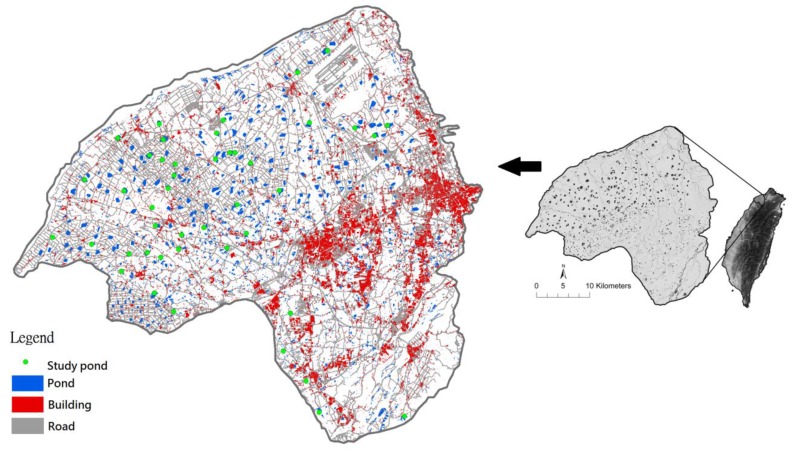
Study area in Taoyuan Tableland, Taiwan.

Birds were surveyed at 45 irrigation ponds in the Taoyuan Tableland, Taiwan, from November 2003 to February 2004 (24°59’ N, 121°18’ E) (Figure 1). Taoyuan receives a substantial amount of precipitation (1849 mm/year); however, the majority of it is unevenly distributed throughout the year [42]. The Taoyuan Tableland, located approximately 40 km southwest of Taipei, occupies an area of 757 km^2^. Taoyuan, which translates to peach garden in English, is situated in a rich agricultural area that contained many peach orchards in the 19th century [42]. Therefore, ponds have been created in this area to store rainwater (10,000 ponds at the peak). The primary function of these ponds is irrigation for agricultural activities [43]. Because urban development has rapidly increased, the Taoyuan metropolitan area now enjoys some of the fastest growth among the six metropolitan areas of Taiwan [42]. Historically, pond sites were constructed on nonpermeable laterite soils containing water. These ponds are also home for birds and other aquatic fauna [44]. Population pressure has contributed to declines in historical areas of farmlands and farm ponds [45]. Losses of farm pond and farmland habitats have severely affected a range of avian communities and other fauna and flora [43].

### 2.2. Sampling

To sample the entire community and account for birds having different degrees of mobility, we used stratified random sampling methods suitable for different habitats within the ponds.

Data regarding birds were recorded using the point count and line transect methods outlined in the Research and Management Techniques for Wildlife and Habitat published by the Wildlife Society [46]. All surveys were conducted by 45 experienced ornithologists commenced at the same time before sunrise, and concluded at 10:00 am on the same day. Each pond was surveyed and coded for numbers of bird species and individuals observed for 30 min by using a point-count approach. No surveys were conducted on extremely windy or rainy days. To minimize the effects of bird-observer-identified bias, groups of three or four observers rotated between ponds. The observers counted birds observed in any habitats. Birds belonging to the families Apodidae (swifts) and Hirundinidae (swallows) were also included based on counting birds in flight and the use of audible/auditory noises. A similar method, namely the line transect method, involves searching or traveling along a given length and recording the number of birds seen and heard within a specified study area. To assess flexibility and field scaling, we surveyed the study area using the extensive network of footpaths that cover it. Therefore, avian observers could reach all irrigation ponds and focus on the water surfaces, mudflats, banks, and vegetation characteristics of the habitats. Thus, stratified random sampling and point counts associated with line transects were used for this avian survey; nine subregions of random samplings were sharply divided and investigated simultaneously to count avian species and individuals to obtain accurate results.

Surveys were conducted during the nonbreeding season of 2003–2004 when deciduous trees were still in leaf in the subtropical region of Taiwan. Birds were surveyed four times in 45 ponds simultaneously over 4 months from November 2003 through February 2004. For this survey, 45 experienced bird observers divided into nine subgroups started their observation before sunrise (07:00) and ended at 10:00 on the same date. Each observer, trained and experienced in identifying ≥ 200 observable species, sampled all pond habitat types equally and rotated into different groups in the subsequent months to avoid sampling bias. Stratified random samples were used for all 45 irrigation ponds selected. Each pond was surveyed and coded for numbers of bird species and individuals within 30 min using a point count approach. Furthermore, totals for the surrounding areas at a 564.19-m basal radius from the pond geometric center (a 100-ha circle) were estimated through the line transect method. The following environmental factors were then considered: pond size (PS); foliage canopy area (FCA); mudflat area (MA); water surface area (WASA); the ratio of farmland (%FARM); the ratio of permanent building area (%BUILD); the ratio of multiple pond areas (%PONDS); the ratio of all watercourse areas covered by rivers, channels, and ditches (%RIVER); and the ratio of all road and trail areas (%ROAD) within a radius of 100 ha from the pond’s geometric center (Table 1). Other variables related to the degree of urbanization (e.g., human density, transportation flow rate, number of automobiles, and number of tall buildings) were not considered because they predominantly influenced species breeding in the urban matrix (e.g., the Eurasian tree sparrow, *Passer montanus*; the light-vented bulbul, *Pycnonotus sinensis*; and the Japanese white-eye, *Zosterops japonicus*) that were recognized as generalists but not specialists within the water surface surrounding farm ponds. In this case, generalists benefit from environments that are spatially heterogeneous, whereas specialists thrive in habitats that are almost homogenous. We, then, used the geographic information system (ArcGIS 9, ESRI, Redlines, CA, USA) to collect the aforementioned data [47].

At the center of each selected pond, a circle of 564.19 m in radius was drawn within an area of 100 ha, and the cover ratios of five major land use types (ponds, watercourses, farmlands, roads, and constructions) and three habitat types (water surfaces, mudflats, and FCAs) were measured. The land use plots were identified based on field surveys, a geographic aerial map (1:5000) of Taiwan, and aerial photographs (1:5000) from 2003. Pond elevations, perimeters, and built-up topologies of waterfronts according to global positioning systems and field surveys were also measured. Information on consolidated areas, as well as the distance from sources that contained the study sites, was derived from the geographic aerial map (1:5000) of Taiwan. All environmental factors formed as patchiness indices were calculated using spatial patterns from aerial photographs (1:5000) using the ArcGIS 9 and FRAGSTATS (Amherst, MA, USA) software programs [48].

PS was determined from the official digital maps of the Department of Land Management, Ministry of the Interior. The MA and WASA, which were considered to be areas for stopovers of migratory species in farm ponds, were measured from aerial photographs (1:5000) and calibrated through field surveys. The FCAs, which might function as corridors or stopovers, were also assessed through contour plots around each pond and measuring the size of wooded areas on the map (1:5000). The same variables were calculated for each of the 45 ponds using ArcGIS 9 and FRAGSTATS. Finally, we analyzed 180 cases (four times each for 45 ponds, degrees of freedom = 179) using SPSS 24.0 (IBM, Armonk, NY, USA) [49].

Cluster analysis was used to identify relationships among the attributes of multivariate samples [49]. The objective of cluster analysis is to group data into clusters, such as elements within guilds [16]. The analysis encompassed a number of algorithms to group birds on the basis of similarities or distance (dissimilarities) [50].

We used the Ward method to merge clusters of species when the nearest neighbor whose distance reached to some groups. The most widespread hierarchical clustering method is the Ward method, which considers the highest similarity [51]. The grouping and value of the error sum of squares (ESS) of the vertical axis at which the mergers occur were clearly illustrated using a dendrogram. The number below each linkage indicates the order in which each fusion occurred. The Ward method was applied with the distance (dissimilarities) index. To avoid loss of information while joining two avian groups, the Ward hierarchical clustering procedure, also known as the method of minimum variance, was adopted to form data partitions to minimize the information loss associated with each grouping. The aim of the Ward procedure is to determine, at each stage, two clusters that merge to provide the minimum increase in the total within the group ESS [52]. Information loss was defined as an ESS criterion:

(1) $$ESS_{i}=\overset{n_{i}}{\underset{j=1}{\sum }}\overset{p}{\underset{k=1}{\sum }}\left(x_{ijk}-\bar{x_{ik}}\right)$$

where $x_{ijk}$ is the multivariate measurement associated with the *j*th item, and $\bar{x_{ik}}$ is the mean of all items. The total within group ESS, $ESS_{i}$, is defined as one stage with k groups, j variables, and $n_{i}$ elements in each group. Therefore, following the summation order, the first sum corresponds to the variability inside a group for a given variable, the second sum is a sum of all variables, and the final sum represents the total variability. The cluster analysis was performed using e SAS 8.0 for Windows [53].

## 3. Results

### 3.1. Classification of Guilds

The survey identified 94 species within 45 point count locations. In Taoyuan, 45 species (48%) were wintering migrants, and 40 species (43%) were breeding residents. Five species were short-transit species (5%) on the farm pond sites, one species (1%) was not present at the site and defined as a “vagrant bird,” and three species (3%) were escaped individuals of domestic species. The total number of species in the winter seasons in the study area varied from a low of 59 (February 2004) to a high of 67 (December 2003). We identified a greater species richness among wintering migrants (48%) compared with permanent residents (45%). On a habitat scale, the individuals we observed most frequently were those in and above the ponds and those on the edge of the ponds.

When surveying the frequencies of occurrence, we identified 10 species that had substantially higher abundances than the other species, accounting for 74% of the total number of individuals (Table 2). Nine of them were categorized as generalist species that could benefit from habitats that are spatially heterogeneous with a higher abundance (see Table A1 in Appendix A). We also identified 84 other species, which accounted for 36% of the total. We detected 23 species with >100 individual birds and 40 species with <10 individual.

Farm ponds were generally associated with the highest number of individuals, with several species restricted to this habitat type. We classified the species into broad categories based on habitat selection, as described by functional terms, guilds, and the groups of species using similar environmental resources within similar periods. Therefore, we calculated the occurrence rate of each species by dividing the number of habitats present by the total number of habitats in each pond (Table 1). This grouping was used for analyses because the source pool sizes were represented by the total number of species in the Taoyuan Tableland.

**Figure 2:**
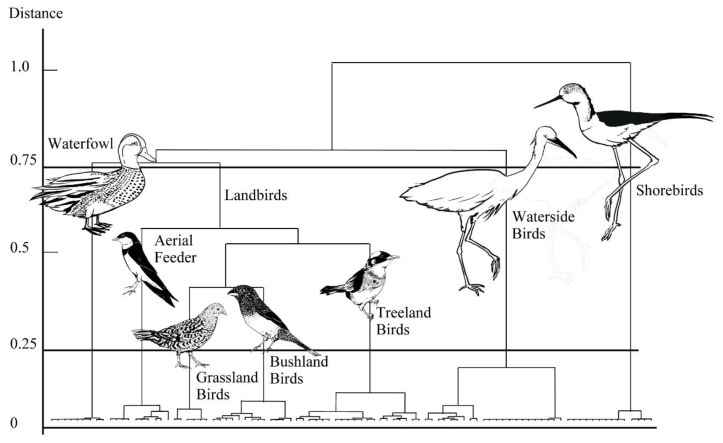
Dendrogram showing the classification of avian data. The Ward method was used with the distance (dissimilarities) index in seven functional groups including 94 species in the study area.

We recorded 94 species when measuring the characteristics of the habitat, which could be broadly classified according to the occurrence rates of where the birds were detected, such as flying overhead, the water’s surface, mudflats, trails and edges, grasslands, bushlands, and woodlands (see Table A1 in Appendix A). The group and value of ESS of the vertical axis merged in Figure 2. The individual numbers detected and species richness of the avian guilds in each month are presented in Table 3.

### 3.2. Habitat Differences with Classification of Guilds

**Figure 3:**
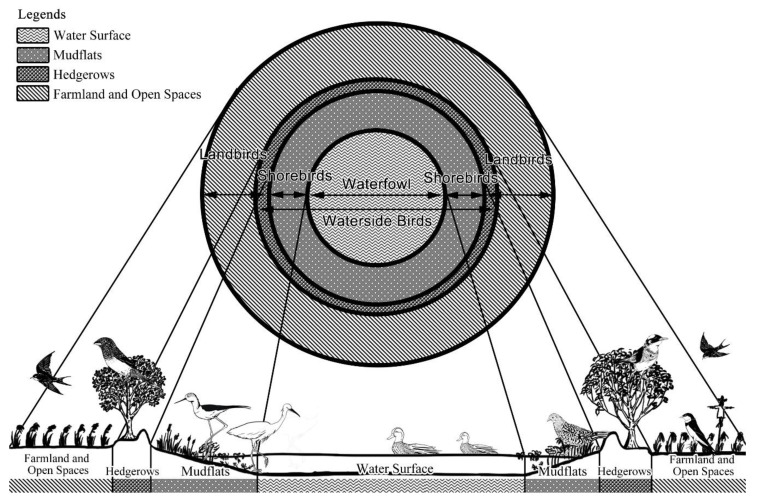
Distribution patterns of relatively dominant bird species in pond habitats successively from the pond’s core to adjacent areas.

According to the dendrogram for the 94 species, we categorized the habitats into seven guilds on the basis of the occurrence rate of avian communities. These guilds were classified according to the dendrogram for similarities between 94 species in the study area (Appendix A and Figure 2). If this classification adopted low similarities (marked to a distance of 0.75), it could be divided into four guilds: waterfowl (9 species), shorebirds (14 species), waterside birds (22 species), and landbirds (49 species). We categorized the likelihood of species occurrence into zones that extended from the pond’s core to its edge. We observed that the (1) interior pond species (i.e., waterfowl and shorebirds), (2) wading species, and (3) external pond species (i.e., landbirds, the species detected in habitats such as grasslands, bushlands, and woodlands) were dominant in their habitats (Figure 3).

### 3.3. Richness and Abundance of Guilds Associated with Environmental Variables

The 14 species of shorebirds demonstrated an increasing trend with increasing MA in a pond (*r* = 0.364, *p* < 0.0001) (Table 4). The selected habitat demonstrated that the guild richness of waterfowl increased with increasing PS (*r* = 0.259, *p* < 0.0001) and MA (*r* = 0.406, *p* < 0.0001). However, these results must be interpreted with caution because the guild contained only nine species. The guild species richness of wading species (22 species) showed a partial negative association with %BUILD (*r* = −0.292, *p* < 0.0001). It also exhibited a partial positive association with PS (*r* = 0.224, *p* < 0.01) and %FARM (*r* = 0.208, *p* < 0.01). Comparison of the wetland birds (i.e., waterfowl, shorebirds, and wading birds) with landbirds (i.e., woodland birds, bushland birds, and grassland birds) revealed that the wetland birds exhibited a stronger correlation with the pond variables than the landbirds did.

The environmental variables that exhibited a good correlation with the abundances of guild species were FCA adjacent to waterfront edges and MA. In general, the individuals of waterfowl species increased with increasing (1) FCA (*r* = 0.503, *p* < 0.0001), (2) %MA (*r* = 0.398, *p* < 0.0001), (3) MA (*r* = 0.347, *p* < 0.0001), and (4) PS (*r* = 0.193, *p* < 0.01). The individuals of shorebird species increased with increasing MA (*r* = 0.291, *p* < 0.0001) and %MA (*r* = 0.367, *p* < 0.0001); however, the number stabilized in the presence of other variables (Table 5). We observed opposite trends for the number of wading species associated with %BUILD (*r* = −0.237, *p* < 0.01). The cumulative individuals of waterside species declined dramatically with the increasing proportion of built-up environment. However, the cumulative individuals of waterside guilds increased with (1) PS (*r* = 0.272, *p* < 0.0001), (2) FCA (*r* = 0.205, *p* < 0.01), (3) %PONDS (*r* = 0.189, *p* < 0.05), (4) %FARM (*r* = 0.169, *p* < 0.05), and (5) WASA (*r* = 0.249, *p* < 0.01). Except in aerial feeders, the guild abundance of landbirds decreased with increasing %ROAD within a radius of 100 ha from the pond’s geometric center in m^2^/ha (*r* = −0.199, *p* < 0.01).

## 4. Discussion

We compared wetland birds (i.e., waterfowl, shorebirds, and wading species) with landbirds (i.e., woodland birds, bushland birds, and grassland birds) to analyze their associations with internal and external pond variables, and the results demonstrated that the individual numbers of waterbirds were more strongly correlated with the pond variables than were the landbirds. Guild analyses further suggested that the principal factor affecting individual birds’ habitat selection was habitat availability. Therefore, the potential for environmental effects on the bird community is high for waterbirds, particularly waterside birds. For individual waterbirds to persist in the agricultural pondscape, fields should have large areas and well-designed neighboring landscapes to support such birds.

Our study results indicated that different avian guilds respond differently to environmental changes. In general, ponds with a larger area for wetland birds and wintering migratory birds could sustain population increases. The associations between population sizes and carrying capacity for individual numbers and species richness of a pond were moderate and slight, respectively, with ponds that support large concentrations of aerial feeders and landbirds as possible exceptions. However, the point at which a pond is regarded as saturated by a single species can be determined by the population sizes of other species wintering on those ponds. Our study results indicated a significant positive correlation between species richness and abundance in several guilds of waterbirds.

In this study, we compared the species-area relationships in ecological groups with similar source pool sizes. These analyses restricted the guild sizes to reduce the possibility of confounding habitat effects. The absence of a habitat of a suitable size was likely a key factor leading to the poor responses of some species in the selection of their wintering ponds. The entire habitats included WASA, MA, and FCA. The waterbirds dependent on large habitat sizes increased in number in more spatially heterogeneous areas, probably as a result of increased safety and food supply. However, other environmental factors, such as the presence of predators and availability of food may also have altered the habitat preferences of waterbirds in the study area.

First, we observed that the species richness and abundance of the wading birds were associated with PS. The total species richness and number of individual waterside birds increased with increasing PS. Second, we observed that individual waterfowl were correlated with the FCA because these specialists or interior species were more sensitive to disturbance than generalists or edge species. Migrants (family Anatidae) and residents (family Podicipedidae) tended to be more sensitive. We discovered that their habitats are far away from the road, farm, or other nonwater regimes. They appeared to be influenced by the level of human disturbance, PS, windbreak size, and pond edge length.

Compared to previous work, we found our study reveal similar result is different pond factors such as depth, size, and vegetation would influence the bird assemblages [54]. Our study is similar in terms of findings with Froneman et al. (2001), they surveyed 59 farm ponds and found 44 bird species in the Elgin and Caledon districts of the Western Cape, South Africa [5]. Compared to their study, we found 94 species in Taoyuan Tableland. Thus, this shows that the farm ponds in Taoyuan are really important for the wintering birds. Besides, the same result is that they found the surface area of the farm ponds as an important variable determining the presence and abundance of many waterbird species [5]. Different from their study, our study included the human structure and found near the buildings and roads, the bird counts and diversity decreased. Anyway, the most important conclusion is that we totally agree artificial waterbodies can play as alternative refuges for biodiversity [55,56,57,58,59].

In our study, we expected to observe the area per se hypothesis within an intermediate range of areas but not at all spatial scales. On a small spatial scale, the species–area relationship is not governed by an equation but is curvilinear on a log-log plot. On a landscape scale, the species-area relationship bends upward toward a limiting slope of unity [60]. We realized that the habitat preferences of birds with different lifestyles must be considered when determining habitat suitability. Most species in small patches associated with the surrounding landscape are generalists, choosing between major habitats and edge habitats. In large patches, the specialists select only interior habitats [61,62]. Therefore, the spatially and taxonomically different species differ in their size [63]. Different avian communities are likely to yield different land-use patches.

In this study, we compared the species-pondscape relationships among ecological groups with their surrounding areas. We restricted these analyses to guild pool sizes to limit the confounding effects of areas. The variables of local determinants of community structures were associated with the amount of farmlands as well as the amount of urban environments. Low-rise residential houses and high-density apartments were observed to affect species richness.

Waterside bird richness displayed a correlation with %BUILD within a radius of 100 ha from the pond’s geometric center because the specialists detected from the pond’s core to the waterfront were more sensitive to anthropogenic disturbance than generalists (i.e., landbirds) were (Table 4). The wading bird species displayed a correlation with %FARM within a radius of 100 ha from the pond’s geometric center. The richness of the waterside bird guild was correlated negatively with increasing urbanization level (indicated by %BUILD); however, the richness correlated positively with increasing green spaces (i.e., farmlands and grasslands). Because we combined environmental factors such as water and edge species of different sizes, foraging modes, and trees from the pond’s core to the waterfront, it is likely that the increase in anthropogenic areas was the principal reason for their decline. In addition, the farmlands, which might translate to greater insect abundance, were strongly correlated with wading bird abundance.

Investigators extensively debated the field domains of the area per se hypothesis and species–habitat hypothesis [64]. However, the generalized principles of ecological designs have yet to be determined, and there is no final consensus on which species-habitat hypothesis is more relevant. As described, birds respond to food and roost sites during habitat selection. The numbers of individual birds of a particular species have been correlated with the requirements for grasslands, mudflats, open shorelines, and canopies or water surfaces for horizontal heterogeneity [65,66]. Therefore, bird-habitat relationships result from the responses of birds using habitats for different activities, such as foraging, molting (i.e., that of the mute swan and greylag goose) [67,68], and roosting in winter. Birds can select pondscape configurations according to their preference.

In this study, the irrigation pond areas were dominated by fields separated by hedgerows and windbreaks, and woods of various sizes were scattered in these areas. The distributions of avian species within such mosaic landscapes were discontinuous, depending on the preferred habitat locations, density-dependent processes, and quality of individual patches. These configurations are surrounded by built-up areas, rivers, roads, and farmlands. In our pondscape evaluations, we used the selected parameters to measure the spatial arrangement of wooded and aquatic landscapes as well as to evaluate the significance of their differences. Previous studies have used these parameters to measure temporal changes in actual landscapes and changes in intensively used landscapes [69,70]. Increasing the pond area increases the pond core area, thereby benefiting specialist species by enhancing the population persistence associated with water depth, water level fluctuation, vegetation, salinity, topography, food type, food accessibility, size, and connectivity [14,15,19]. Our results indicated a requirement for relevant conservation scenarios to focus on vulnerable sites, which might be targeted for enlargement by habitat creation at their woody edges, on the basis that large pond habitats are broadly beneficial for biodiversity. Thus, we suggest a study on yearly or interannual variability of wintering waterbirds to analyze the effect of habitat changes on birds.

## 5. Conclusions

To construct waterbird refuges in Taoyuan, Taiwan, for securing habitats for wintering waterbirds in the areas of anthropogenic influence, existing and potential irrigation ponds must be identified. Pond conservation for bird refuges is difficult because of increasing urban development, which exerts pressure on avian communities [43,71]. Changes in land use [72], particularly consolidating farming practices with urban construction, affect avian communities in ponds. Evaluating the pond habitats of winter birds might provide useful information for simulating the pond environment to identify the criteria of their habitat selection behaviors. On the habitat and landscape scales, specific selection according to avian assemblages is required for identifying large areas within fields to support various waterbird species.

The number of farm ponds in Taoyuan Tableland is decreasing, thus providing evidence of the negative effects of landscape quality on the avian distribution and species in these fragmented habitats. In our avian population studies, the functional groups associated with pondscape configurations provided an effective tool for determining linear relationships based on the concept of landscape ecology. We used theoretical models to quantify the influence of landforms on avian groups. Our study provided substantial evidence that artificial ponds also influence wintering waterbirds. The final results regarding ponds may aid stakeholders and land managers to identify areas for the establishment of large-scale solar facilities in pond areas for superior management of wildlife. In conclusion, our study provided a comprehensive view of farm pond-bird interaction in Taoyuan Tableland, thus aiding decision-makers in enacting policies beneficial to both humans and the environment.

The results related to the condition of the birds at farm ponds in Taoyuan Tableland from 2003 to 2004. We, therefore, can compare the current condition of birds with that of 15 years ago for a better understanding of the relationships of human activities and wildlife biodiversity.

## Figures and Tables

**Table 1:** Definition and description of metrics used in patch analysis of factors that influence bird communities.

Item Acronym	Pondscape Variables (Metrics/Units)	Description
PS ^b^	Pond size ^1^	Pond size (m^2^)
FCA ^b^	Foliage canopy area next to waterfront edge of a pond (m^2^) ^1^	Boundary delineation of disturbance
MA ^a,b^	Mudflat area in a pond (m^2^) ^1^	Boundary delineation of disturbance
WASA ^a,b^	Water surface area in a pond (m^2^) ^1^	Boundary delineation of disturbance
%FCA ^b^	FCA ÷ PS ^1^	
%MA ^b^	MA ÷ PS ^1^	
%FARM ^b^	The ratio of farmland areas within a radius of 100 ha from the pond’s geometric center (m^2^)/ha ^1^	Pondscape isolation or connectivity from the center of each selected pond
%BUILD ^b^	The ratio of permanent building areas within a radius of 100 ha from the pond’s geometric center (m^2^)/ha ^1^	Pondscape isolation from center of each selected pond
%PONDS ^b^	The ratio of multiple pond areas within a radius of 100 ha from the pond’s geometric center (m^2^)/ha ^1^	Pondscape connectivity from the center of each selected pond
%RIVER ^b^	The ratio of all watercourse areas covered by rivers, creeks, channels, and ditches within a radius of 100 ha from the pond’s geometric center (m^2^)/ha ^1^	Pondscape connectivity from the center of each selected pond
%ROAD ^b^	The ratio of all road and trail areas within a radius of 100 ha from the pond’s geometric center (m^2^)/ha ^1^	Pondscape isolation from the center of each selected pond

^1^ Mean values are expressed in percent. The different land use types were measured as a percentage area of a circle with an area of 100 ha (radius = 564.19 m) centered on each of the survey ponds (*n* = 45). The range of the percentage area of each land use type is also given. ^a^ Variable based on field measurements. ^b^ Variable based on the GIS.; PS: pond size; FCA: foliage canopy area; MA: mudflat area; WASA: water surface area; %FARM: the ratio of farmland; %PONDS: the ratio of permanent building area; the ratio of multiple pond areas; %RIVER: the ratio of all watercourse areas covered by rivers, channels, and ditches; %ROAD: the ratio of all road and trail areas, within a radius of 100 ha from the pond’s geometric center.

**Table 2:** Identification of nine generalist species, which were greater in number than other species and accounted for 74% of all species abundance (only little grebe, *Tachybaptus ruficollis*, is not a generalist species).

Species	Occurrence Frequency	Occurrence Rate	Type of the Birds
Black-crowned night heron (*Nycticorax nycticorax*)	2363	15.7%	Resident
Little egret (*Egretta garzetta*)	1883	12.5%	Resident
Grey heron (*Ardea cinerea*)	1829	12.2%	Wintering visitor
Light-vented bulbul (*Pycnonotus sinensis*)	1575	10.5%	Resident
Eurasian tree sparrow (*Passer montanus*)	1125	7.7%	Resident
Great egret (*Casmerodius alba*)	726	4.8%	Wintering visitor
Red-collared dove (*Streptopelia tranquebarica*)	509	3.4%	Resident
Japanese white-eye (*Zosterops japonica*)	504	3.3%	Resident
Little ringed plover (*Charadrius dubius*)	316	2.1%	Wintering visitor
Little grebe (*Tachybaptus ruficollis*)	304	2%	Resident
Total	11,134	74.2%	

**Table 3:** Individual numbers and species richness of avian guilds in each month.

Classification	November 2003	December 2003	January 2004	February 2004
Aerial feeder	96 (5)	248 (7)	90 (6)	79 (4)
Waterfowl	85 (6)	209 (6)	157 (7)	132 (5)
Shorebirds	240 (6)	261 (10)	212 (10)	94 (6)
Waterside birds	2192 (10)	1776 (14)	1775 (11)	1465 (15)
Grassland birds	31 (4)	127 (3)	9 (2)	12 (4)
Bushland birds	233 (11)	213 (9)	354 (9)	296 (8)
Woodland birds	844 (18)	1438 (18)	1303 (17)	1082 (17)
Individual no. (species richness)	3721 (60)	4272 (67)	3900 (62)	3160 (59)

The individual numbers have been detected in each group; the value within parentheses. Brackets indicate the species richness.

**Table 4:** Pearson product–moment correlation coefficients indicating the coefficients between guild species richness and pondscape variables.

Classification	Aerial Feeders	Waterfowl	Shorebirds	Waterside Birds	Landbirds ^1^
PS	−0.073	0.259 **	0.102	0.224 **	−0.100
FCA	−0.006	0.305 **	0.140	0.226 **	0.078
MA	−0.076	0.406 **	0.364 **	0.021	−0.108
WASA	−0.048	0.112	−0.043	0.236 **	−0.063
%FCA	0.022	−0.026	0.043	−0.185 **	−0.057
%MA	−0.033	0.387 **	0.408 **	0.063	−0.094
%FARM	−0.039	−0.104	0.006	0.208 **	0.044
%BUILD	0.109	0.016	−0.059	−0.292 **	−0.028
%PONDS	−0.197 **	0.162 *	0.133	0.199 **	−0.145
%RIVER	0.172 *	0.163 *	0.039	−0.111	0.102
%ROAD	0.020	−0.057	−0.122	−0.052	−0.135

^1^ Landbirds: This group included grassland birds, bushland birds, and woodland birds, but not aerial feeders in this case. * The correlation is statistically significant at the 0.05 significance level, two-tailed. ** The correlation is statistically significant at the 0.01 significance level, two-tailed.

**Table 5:** Pearson product–moment correlation coefficients indicating the correlation between guild species individuals and pondscape variables.

Classification	Aerial Feeders	Waterfowl	Shorebirds	Waterside Birds	Landbirds ^1^
PS	−0.059	0.193 **	0.091	0.272 **	−0.059
FCA	−0.002	0.503 **	0.007	0.205 **	0.140
MA	−0.054	0.347	0.291 **	0.114	−0.084
WASA	−0.041	0.064	−0.024	0.249 **	−0.029
%FCA	−0.034	−0.046	0.005	−0.120	−0.018
%MA	−0.021	0.398 **	0.367 **	0.184 *	−0.058
%FARM	−0.091	−0.005	0.011	0.169 *	0.076
%BUILD	0.099	−0.069	−0.036	−0.237 **	−0.010
%PONDS	−0.093	0.171 *	0.075	0.189 *	−0.075
%RIVER	0.256 **	0.095	−0.018	−0.117	−0.034
%ROAD	0.035	−0.151 *	−0.064	−0.110	−0.199 **

^1^ Landbirds: This group included grassland birds, bushland birds, and woodland birds, but not aerial feeders in this case. * The correlation is statistically significant at the 0.05 significance level, two-tailed. ** The correlation is statistically significant at the 0.01 significance level, two-tailed.

## Appendix A

**Table A1:** List of birds detected in Taoyuan farm ponds and their occurrence rate in different habitats.

ID	Code	Common Name	Scientific Name	Air	Waters	Mudflats	Trails	Grasslands	Bushlands	Woodlands	Avian Guilds
1	1402	Northern pintail	*Anas acuta*	0.00	1.00	0.00	0.00	0.00	0.00	0.00	Waterfowl **
2	1403	Northern shoveller	*Anas clypeata*	0.00	1.00	0.00	0.00	0.00	0.00	0.00	Waterfowl **
3	1404	Common teal	*Anas crecca*	0.00	1.00	0.00	0.00	0.00	0.00	0.00	Waterfowl **
4	1408	Eurasian wigeon	*Anas penelope*	0.00	1.00	0.00	0.00	0.00	0.00	0.00	Waterfowl **
5	1410	Spot-billed duck	*Anas poecilorhyncha*	0.00	1.00	0.00	0.00	0.00	0.00	0.00	Waterfowl **
6	1419	Common pochard	*Aythya ferina*	0.00	1.00	0.00	0.00	0.00	0.00	0.00	Waterfowl **
7	1523	Black-eared kite	*Milvus migrans lineatus*	1.00	0.00	0.00	0.00	0.00	0.00	0.00	Aerial Feeder **
8	2102	Common coot	*Fulica atra*	0.00	0.00	1.00	0.00	0.00	0.00	0.00	Shorebird **
9	2106	Ruddy-breasted crake	*Porzana fusca*	0.00	0.00	1.00	0.00	0.00	0.00	0.00	Shorebird **
10	2608	Grey-headed lapwing	*Vanellus cinereus*	0.00	0.00	1.00	0.00	0.00	0.00	0.00	Shorebird **
11	2609	Pacific golden plover	*Pluvialis fulva*	0.00	0.00	1.00	0.00	0.00	0.00	0.00	Shorebird **
12	2738	Common redshank	*Tringa totanus*	0.00	0.00	1.00	0.00	0.00	0.00	0.00	Shorebird **
13	2801	Black-winged stilt	*Himantopus himantopus*	0.00	0.00	1.00	0.00	0.00	0.00	0.00	Shorebird **
14	2802	Pied avocet	*Recurvirostra avosetta*	0.00	0.00	1.00	0.00	0.00	0.00	0.00	Shorebird **
15	4002	Fork-tailed swift	*Apus pacificus*	1.00	0.00	0.00	0.00	0.00	0.00	0.00	Aerial Feeder **
16	4903	Striated swallow	*Hirundo striolata*	1.00	0.00	0.00	0.00	0.00	0.00	0.00	Aerial Feeder **
17	4905	Plain sand martin	*Riparia paludicola*	1.00	0.00	0.00	0.00	0.00	0.00	0.00	Aerial Feeder **
18	6701	Red-throated pipit	*Anthus cervinus*	0.00	0.00	1.00	0.00	0.00	0.00	0.00	Shorebird **
19	2735	Common greenshank	*Tringa nebularia*	0.00	0.00	0.97	0.03	0.00	0.00	0.00	Shorebird
20	1421	Greater scaup	*Aythya marila*	0.00	0.95	0.00	0.00	0.00	0.00	0.05	Waterfowl
21	205	Little grebe	*Tachybaptus ruficollis*	0.00	0.94	0.00	0.06	0.00	0.00	0.00	Waterfowl
22	2104	Common moorhen	*Gallinula chloropus*	0.00	0.00	0.89	0.08	0.03	0.00	0.01	Shorebird
23	1409	Mallard	*Anas platyrhynchos*	0.00	0.88	0.00	0.12	0.00	0.00	0.00	Waterfowl
24	4001	House swift	*Apus nipalensis*	0.81	0.00	0.00	0.19	0.00	0.00	0.00	Aerial Feeder
25	2731	Wood sandpiper	*Tringa glareola*	0.00	0.00	0.80	0.20	0.00	0.00	0.00	Shorebird
26	3207	Common black-headed gull	*Larus ridibundus*	0.75	0.00	0.00	0.25	0.00	0.00	0.00	Aerial Feeder
27	2601	Kentish plover	*Charadrius alexandrinus*	0.00	0.00	0.73	0.27	0.00	0.00	0.00	Shorebird
28	4904	Pacific swallow	*Hirundo tahitica*	0.73	0.00	0.00	0.19	0.01	0.00	0.07	Aerial Feeder
29	2611	Northern lapwing	*Vanellus vanellus*	0.00	0.00	0.72	0.28	0.00	0.00	0.00	Shorebird
30	2603	Little ringed plover	*Charadrius dubius*	0.00	0.00	0.77	0.17	0.06	0.00	0.00	Shorebird *
31	1108	Great egret	*Casmerodius alba*	0.00	0.59	0.00	0.34	0.00	0.00	0.07	Waterside bird *
32	4101	Common kingfisher	*Alcedo atthis*	0.55	0.00	0.00	0.25	0.02	0.11	0.08	Aerial Feeder
33	4902	Barn swallow	*Hirundo rustica*	0.53	0.00	0.00	0.47	0.00	0.00	0.00	Aerial Feeder
34	1706	Common kestrel	*Falco tinnunculus*	0.50	0.00	0.00	0.00	0.00	0.00	0.50	Aerial Feeder
35	1111	Intermediate egret	*Mesophoyx intermedia*	0.00	0.47	0.00	0.45	0.00	0.00	0.08	Waterside bird
36	1110	Little egret	*Egretta garzetta*	0.00	0.54	0.00	0.28	0.01	0.04	0.13	Waterside bird *
37	2733	Common sandpiper	*Tringa hypoleucos*	0.00	0.00	0.43	0.51	0.04	0.03	0.00	Waterside bird
38	1101	Grey heron	*Ardea cinerea*	0.00	0.34	0.00	0.46	0.02	0.01	0.17	Waterside bird *
39	901	Common cormorant	*Phalacrocorax carbo*	0.00	0.32	0.00	0.64	0.00	0.00	0.04	Waterside bird
40	2703	Dunlin	*Calidris alpina*	0.00	0.00	0.28	0.72	0.00	0.00	0.00	Waterside bird
41	1121	Black-crowned night heron	*Nycticorax nycticorax*	0.00	0.23	0.00	0.59	0.01	0.07	0.10	Waterside bird *
42	6707	White wagtail	*Motacilla alba*	0.00	0.22	0.00	0.27	0.51	0.00	0.00	Grassland bird
43	5410	Black-billed magpie	*Pica pica*	0.20	0.00	0.00	0.07	0.00	0.10	0.63	Woodland bird
44	1601	Osprey	*Pandion haliaetus*	0.17	0.00	0.00	0.00	0.00	0.00	0.83	Woodland bird
45	5914	Rufous-capped babbler	*Stachyris ruficeps*	0.00	0.14	0.00	0.14	0.00	0.00	0.71	Woodland bird
46	3509	Red-collared dove	*Streptopelia tranquebarica*	0.00	0.13	0.00	0.19	0.01	0.02	0.66	Woodland bird *
47	6710	Yellow wagtail	*Motacilla flava*	0.00	0.11	0.00	0.22	0.64	0.02	0.01	Grassland bird
48	5403	Large-billed crow	*Corvus macrorhynchos*	0.00	0.11	0.00	0.28	0.00	0.00	0.60	Woodland bird
49	6708	Grey wagtail	*Motacilla cinerea*	0.00	0.11	0.00	0.63	0.22	0.03	0.01	Waterside bird
50	5913	Steak-breasted; Scimitar babbler	*Pomatorhinus ruficollis*	0.00	0.05	0.00	0.16	0.00	0.68	0.11	Bushland bird
51	5103	Black drongo	*Dicrurus macrocercus*	0.00	0.05	0.00	0.29	0.19	0.14	0.34	Woodland bird
52	6313	Daurian redstart	*Phoenicurus auroreus*	0.00	0.04	0.00	0.21	0.38	0.13	0.25	Woodland bird
53	6422	Plain prinia	*Prinia inornata*	0.00	0.04	0.00	0.36	0.08	0.47	0.05	Bushland bird
54	7201	Japanese white-eye	*Zosterops japonica*	0.00	0.03	0.00	0.03	0.01	0.10	0.84	Woodland bird *
55	3507	Spotted dove	*Streptopelia chinensis*	0.00	0.02	0.00	0.21	0.08	0.00	0.68	Woodland bird
56	1105	Cattle egret	*Bubulcus ibis*	0.00	0.02	0.00	0.21	0.00	0.01	0.77	Woodland bird
57	7012	White-vented myna	*Acridotheres grandis*	0.00	0.02	0.00	0.53	0.00	0.00	0.45	Woodland bird
58	6003	Light-vented bulbul	*Pycnonotus sinensis*	0.00	0.01	0.00	0.14	0.02	0.14	0.69	Woodland bird *
59	7601	Eurasian tree sparrow	*Passer montanus*	0.00	0.01	0.00	0.23	0.04	0.21	0.51	Woodland bird *
60	1119	Yellow bittern	*Ixobrychus sinensis*	0.00	0.00	0.00	0.00	1.00	0.00	0.00	Grassland bird
61	1802	Chinese bamboo partridge	*Bambusicola thoracica*	0.00	0.00	0.00	0.00	1.00	0.00	0.00	Grassland bird **
62	2101	White-breasted waterhen	*Amaurornis phoenicurus*	0.00	0.00	0.00	1.00	0.00	0.00	0.00	Waterside bird **
63	2707	Rufous-necked stint	*Calidris ruficollis*	0.00	0.00	0.00	1.00	0.00	0.00	0.00	Waterside bird **
64	2709	Temminck’s stint	*Calidris temminckii*	0.00	0.00	0.00	1.00	0.00	0.00	0.00	Waterside bird **
65	2713	Common snipe	*Gallinago gallinago*	0.00	0.00	0.00	1.00	0.00	0.00	0.00	Waterside bird **
66	2729	Grey-tailed tattler	*Tringa brevipes*	0.00	0.00	0.00	1.00	0.00	0.00	0.00	Waterside bird **
67	3508	Eastern turtle dove	*Streptopelia orientalis*	0.00	0.00	0.00	0.05	0.00	0.03	0.91	Woodland bird
68	3512	White-bellied green pigeon	*Treron sieboldii*	0.00	0.00	0.00	0.00	0.00	0.00	1.00	Woodland bird **
69	3601	Lesser coucal	*Centropus bengalensis*	0.00	0.00	0.00	1.00	0.00	0.00	0.00	Waterside bird **
70	4501	Black-browed barbet	*Megalaima oorti*	0.00	0.00	0.00	0.00	0.00	0.00	1.00	Woodland bird **
71	5407	Grey treepie	*Dendrocitta formosae*	0.00	0.00	0.00	0.00	0.43	0.43	0.14	Bushland bird
72	5502	Vinous-throated parrotbill	*Paradoxornis webbianus*	0.00	0.00	0.00	0.21	0.00	0.65	0.14	Bushland bird
73	6002	Black bulbul	*Hypsipetes leucocephalus*	0.00	0.00	0.00	1.00	0.00	0.00	0.00	Waterside bird **
74	6307	Siberian rubythroat	*Luscinia calliope*	0.00	0.00	0.00	0.33	0.00	0.67	0.00	Bushland bird
75	6317	Orange-flanked bush-robin	*Tarsiger cyanurus*	0.00	0.00	0.00	0.00	0.00	1.00	0.00	Bushland bird **
76	6321	Brown-headed thrush	*Turdus chrysolaus*	0.00	0.00	0.00	1.00	0.00	0.00	0.00	Waterside bird **
77	6325	Dusky thrush	*Turdus naumanni*	0.00	0.00	0.00	1.00	0.00	0.00	0.00	Waterside bird **
78	6402	Great reed warbler	*Acrocephalus arundinaceus*	0.00	0.00	0.00	0.00	1.00	0.00	0.00	Grassland bird **
79	6406	Japanese bush warbler	*Cettia diphone*	0.00	0.00	0.00	0.33	0.00	0.00	0.67	Woodland bird
80	6407	Brownish-flanked bush warbler	*Cettia fortipes*	0.00	0.00	0.00	1.00	0.00	0.00	0.00	Waterside bird **
81	6410	Zitting cisticola	*Cisticola juncidis*	0.00	0.00	0.00	0.00	0.00	1.00	0.00	Bushland bird **
82	6421	Yellow-bellied prinia	*Prinia flaviventris*	0.00	0.00	0.00	0.22	0.12	0.56	0.10	Bushland bird
83	6703	Olive-backed pipit	*Anthus hodgsoni*	0.00	0.00	0.00	1.00	0.00	0.00	0.00	Waterside bird **
84	6902	Brown shrike	*Lanius cristatus*	0.00	0.00	0.00	0.22	0.03	0.39	0.36	Woodland bird
85	6904	Long-tailed shrike	*Lanius schach*	0.00	0.00	0.00	0.00	0.00	0.63	0.38	Bushland bird
86	7001	Crested myna	*Acridotheres cristatellus*	0.00	0.00	0.00	0.89	0.00	0.00	0.11	Waterside bird
87	7002	Common myna	*Acridotheres tristis*	0.00	0.00	0.00	0.31	0.00	0.23	0.46	Woodland bird
88	7005	White-cheeked starling	*Sturnus cineraceus*	0.00	0.00	0.00	0.00	1.00	0.00	0.00	Grassland bird **
89	7007	White-shouldered starling	*Sturnus sinensis*	0.00	0.00	0.00	0.00	0.00	1.00	0.00	Woodland bird **
90	7008	Red-billed starling	*Sturnus sericeus*	0.00	0.00	0.00	1.00	0.00	0.00	0.00	Waterside bird **
91	7302	Scaly-breasted munia	*Lonchura punctulata*	0.00	0.00	0.00	0.15	0.26	0.48	0.11	Bushland bird
92	7303	White-rumped munia	*Lonchura striata*	0.00	0.00	0.00	0.00	0.14	0.86	0.00	Bushland bird
93	7511	Black-faced bunting	*Emberiza spodocephala*	0.00	0.00	0.00	0.20	0.20	0.54	0.07	Bushland bird
94	9902	Rose-ringed parakeet	*Psittacula krameri*	0.00	0.00	0.00	0.50	0.00	0.00	0.50	Woodland bird

Note: This study has been used in cluster analysis to identify bird guilds and simply list the birds recorded, organize them into well-known groups, and indicate with asterisks those that are understood to be generalists (*) or specialists (**). In this case, ecological specialists (**) thrive in a nearly homogenous habitat with a 100% occurrence rate.
